# HIV testing at birth: Are we getting it right?

**DOI:** 10.4102/sajhivmed.v20i1.951

**Published:** 2019-06-27

**Authors:** Chanté Bisschoff, Jasmine Coulon, Ziva Isaacs, Lavinia van der Linde, Linley Wilson, Riana van Zyl, Gina Joubert

**Affiliations:** 1 Department of Paediatrics and Child Health, Faculty of Health Sciences, University of the Free State, Bloemfontein, South Africa; 2 Department of Biostatistics, Faculty of Health Sciences, University of the Free State, Bloemfontein, South Africa

**Keywords:** Birth HIV PCR testing, Follow-up testing, Prevention of mother-to-child Transmission, National guidelines, Documentation, Communication

## Abstract

**Background:**

Birth polymerase chain reaction (PCR) testing improves early detection of HIV and allows for early treatment initiation. National guidelines exist, but it is unknown whether these are being implemented correctly.

**Objectives:**

To determine whether HIV-exposed infants at the Mangaung University Community Partnership Programme Community Health Centre (MUCPP CHC) received PCR tests at birth, if HIV-positive infants were initiated on treatment, if follow-up dates were scheduled and the percentage of mothers or caregivers who returned to collect the results.

**Methods:**

The study was a retrospective descriptive file audit (1304 files) of births from 01 January to 31 December 2016 at MUCPP CHC. The study sample was 428 infants born to HIV-positive mothers. The birth register was used to collect the infants’ HIV PCR test barcodes. The birth and 10-week PCR results were retrieved from an electronic database at the Virology Department, University of the Free State.

**Results:**

In total, 375 infants received a birth PCR test (87.6%) of which 4 (1.1%) tested HIV positive and 327 (87.2%) negative. Follow-up tests were not scheduled. However, 145 (44.3%) HIV-negative infants returned for a 10-week test. Irrespective of the PCR birth result, 157 (36.7%) infants were brought for a 10-week follow-up test at which time 3 (1.9%) tested positive and 151 (96.2%) negative.

**Conclusion:**

The majority of HIV-exposed infants received a PCR test at birth; however, the clinic is below the national target (90%) for HIV testing. A record-keeping system of infants’ visits does not exist at MUCPP CHC, making it impossible to determine whether HIV-positive infants were started on antiretroviral treatment.

## Introduction

The risk of an infant being infected with HIV during pregnancy, delivery or breastfeeding can be reduced to 5% or less if the mother is on antiretroviral treatment (ART).^1^ The highest mortality in an infant who has acquired HIV is between the ages of 6 weeks to 4 months; therefore, early infant diagnosis is imperative in identifying the status of the infant for early introduction of treatment.^1,2,3,4^ A South African trial conducted in HIV-positive infants showed improved short-term neurodevelopmental outcomes because of early initiation of ART, in comparison to infants for whom treatment was delayed.^5^ Violari et al.^6^ found that infant mortality was reduced by 76% and HIV progression by 75% when infants infected with HIV were diagnosed and placed on ART before 12 weeks of age.

The South African National Department of Health released the 2015 ‘National consolidated guidelines for prevention of mother-to-child transmission (PMTCT) and the management of HIV in children, adolescents and adults’^7^ which makes provision for birth HIV polymerase chain reaction (PCR) testing for all HIV-exposed infants. The reason for the birth PCR was to promote immediate ART and linkage to care. The infants must receive immediate ART if they test positive with the first PCR test, and a second PCR test must be performed as a confirmatory test within 1 week after the first PCR test.^7,8^ At 10 weeks, all HIV-exposed but uninfected infants will have a repeat PCR. This is performed 4 weeks after the cessation of nevirapine administration. If the infant is still on nevirapine prophylaxis and the PCR test is performed, there is a possibility of a false negative or indeterminate result.^9^ A definite diagnosis of HIV in infants younger than 18 months of age needs two positive PCR results.^7^

The Mangaung University Community Partnership Programme Community Health Centre (MUCPP CHC) in the Free State is a large primary health care facility, which consists of an antenatal clinic, a maternity ward and a paediatric clinic, and provides comprehensive HIV management for adults and children. Only uncomplicated normal vaginal deliveries (NVD) are performed on site, while complicated deliveries and caesarean sections are referred to the Pelonomi Academic Hospital for specialist management. As MUCPP CHC uses the 2015 national guidelines,^7^ the authors were interested in how strictly these guidelines were adhered to.

### Aim

The aim of this study was to determine whether the neonatal component of the 2015 ‘National consolidated guidelines for PMTCT of HIV and the management of HIV in children, adolescents and adults’^7^ was being implemented in the management of HIV-exposed infants on a PHC level at MUCPP CHC in Bloemfontein, Free State.

Specific objectives included the following:

- Whether all HIV-exposed infants received an HIV PCR test at birth
- Whether the infants who tested HIV-positive were initiated on ART within a week of diagnosis
- Whether a follow-up date for the 10-week follow-up test was given to the mother or caregiver of the infant who tested HIV-negative at birth
- The percentage of mothers or caregivers who returned to collect the birth HIV PCR results.

## Methods

### Study design and sampling

This was a retrospective descriptive file audit. The study population included all mothers who gave birth to a live baby via NVD at MUCPP CHC from 01 January 2016 to 31 December 2016.

### Measurement

The mothers’ files are kept at the maternity ward of MUCPP CHC. The researchers counted every file from 01 January to 31 December 2016. The mothers’ files were used to identify whether they were HIV-positive or not and whether they had an uncomplicated NVD. The mother’s age, parity and gravidity, HIV status and whether she was on ART were captured on a data sheet.

The 2016 birth register was used to collect the HIV-exposed infants’ PCR barcodes and any information that was missing from the mothers’ files, for example whether the mother was on ART. The researchers collected the HIV PCR results using these PCR barcodes for the birth tests, while the date of birth and surname of the infant were used to obtain the HIV PCR test results, including the 10-week HIV PCR test results. This was performed using the National Health Laboratory System (NHLS) electronic database at the Department of Virology, University of the Free State (UFS).

### Pilot study

The pilot study was conducted at MUCPP CHC. The first 10 files of HIV-positive mothers of January 2016 with an uncomplicated NVD were selected. During the pilot study, it became clear that the information to achieve our objectives was not noted in the patient files and the methodology had to be amended to obtain PCR results from the Virology database. It was thus not possible to determine whether infants who tested HIV-positive were initiated on ART within a week of diagnosis, whether follow-up dates were given to mothers and whether mothers collected birth PCR results. Changes were made to the data sheet, which included removing the fields for the infant’s weight, height and if the infant was on medication, because this information was found to be unavailable and did not contribute to the study aim. Demographic information, hospital number, the option of ‘unknown’, the clinic’s name for antenatal care and dates of all the HIV PCR tests were added. The information gathered from the pilot study was included in the main study.

### Analysis of data

The researchers entered the data into Microsoft Excel. Data were analysed by the Department of Biostatistics, Faculty of Health Sciences, University of Free State. Numerical variables were summarised by medians and ranges because of skewed distributions. Categorical variables were summarised by frequencies and percentages.

The 10-week HIV PCR test results were categorised as follows:

- Before 10 weeks (1–55 days)
- At 10 weeks (56–89 days)
- After 3 months (90+ days)

## Ethical consideration

The protocol was approved by the Health Sciences Research Ethics Committee (HSREC) of UFS [HSREC-S 06/2017] and the Free State Department of Health. The data were handled confidentially, and each patient file received a unique study number. Access to the information on the NHLS electronic database was granted by the Head of the Department of Virology, UFS.

## Results

The file review showed that 1304 live babies were born via uncomplicated NDV during 2016. There were, however, 20 duplicate data entries and 10 cases were thus removed. Eight incorrect entries were also removed leaving 1286 patient files. The number of deliveries per month ranged between 65 (minimum, recorded in July) and 151 (maximum, recorded in August). The study sample included 428 (33.3%) babies born to HIV-positive mothers. Just over half of these babies were male (*n* = 214, 50.4%).

The median age of the HIV-positive mothers was 23.3 years (range: 16.4–44.5 years). Most mothers were between the ages of 20 and 35 years (82.6%). There were 31 (7.3%) teenage pregnancies (mothers < 18 years old) and 43 (10.1%) pregnancies in mothers aged 35 years and older. Nearly half the mothers had been pregnant at least twice before (parity ≥ 2, 48.5%; gravidity ≥ 3, 45.5%). The ART details were recorded for only 410 of the mothers of which 361 (88.0%) mothers were on ART.

**FIGURE 1:**
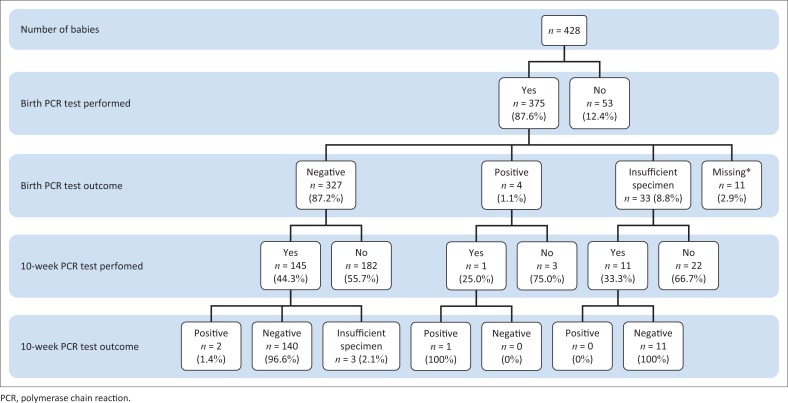
Birth and 10-week polymerase chain reaction testing of HIV-exposed infants.

Figure 1 is a schematic representation of the HIV-exposed infants and their PCR testing and outcomes at birth and 10 weeks.

The majority of HIV-exposed infants (87.6%) received an HIV-1 PCR test at birth, of whom 87.2% tested HIV-negative. When looking at month-specific percentages, during November, only 47.9% (23/48) of HIV-exposed infants received birth PCR tests. Instead, a viral load test was performed on the mother. There was no outcome recorded for 11.7% of all infants tested at birth, because of either insufficient specimen or missing results. Of the 22 infants born in July, 16 (72.7%) had an insufficient specimen. This is 48.5% of the total of 33 insufficient specimens at birth. Of these 33 infants, only a third were retested at 10 weeks.

Of the 34 babies born to teenage mothers, 82.4% had an HIV-1 PCR test at birth. The birth PCR results were conclusive in 24 babies of whom 2 (7.7%) tested positive. Of the 43 infants born to mothers aged 35 and older, 90.7% had an HIV-1 PCR test at birth, but none of these babies had a positive birth PCR test.

Of the infants who tested HIV-negative at birth, 44.3% had a repeat PCR at 10 weeks, of whom 1.4% tested HIV-positive. Irrespective of the HIV PCR test outcome at birth (positive, negative or insufficient specimen), 43.1% of the infants (157/364) returned for a 10-week follow-up visit of whom 3 (1.9%) tested positive, 151 (96.2%) tested negative and 3 (1.9%) had an insufficient specimen for a result. The median number of days for the 10-week PCR testing was 73 days, and the mode was 70 days.

Of the 375 babies who had a birth PCR, 57.3% (*n* = 215) had a follow-up test at any date.

Twenty mothers or caregivers returned with their infants for follow-up testing before the 10-week time frame (i.e. ≤ 55 days after the birth HIV PCR test). The median number of days was 42 days (range: 7–46 days). Eighteen infants tested negative, and two infants had an insufficient specimen. Two infants were tested before 10 weeks and again at 10 weeks. One infant tested negative on both occasions. The second infant had an insufficient specimen before 10 weeks and tested negative at 10 weeks.

Fifty infants were tested only after 3 months (i.e. 90 or more days after the birth HIV PCR test).The median number of days was 119 days (range: 90–441 days). The PCR outcome for 49 of the infants was negative, and one infant had an insufficient specimen. Four infants were tested before 10 weeks and again after 3 months. All four infants tested negative at both dates.

Nevirapine was given to 427 (99.8%) infants with one file having no record of this.

## Discussion

A third (33.3%) of the mothers who gave birth at MUCPP CHC during 2016 were HIV-positive. Among these HIV-positive mothers, there were 7.3% teenage pregnancies and 10.1% pregnancies to women over the age of 35 years, also classified as post-maternal. Pregnancies in either very young women (teenagers younger than 18 years) or women older than 35 years (advanced maternal age) are considered to be high-risk pregnancies.^10^ Especially, teenage pregnancy and multiparity carry an increased risk of maternal HIV infection and vertical transmission.

The percentage of HIV-positive mothers on ART at MUCPP CHC is comparable to the 95.0% of pregnant HIV-positive women in South Africa on ART in 2016.^11^

The results showed that 87.6% (375/428) of HIV-exposed infants born at MUCPP CHC during 2016 received a birth PCR test. The national target for HIV testing in the general population in South Africa is 90.0%.^7^ The 2015 national guidelines^7^ also state that all HIV-exposed infants should receive a birth PCR test as early diagnosis is essential to decrease HIV-related deaths in infants.

It is possible that not all nurses were aware of the 2015 national guidelines^7^ and they still follow the 2010 national guidelines^12^ in which the first HIV PCR test is performed only at 6 weeks of age while the mother receives a viral load test of delivery. This practice seemed to be employed by staff working at the clinic during November. Different staff members also seem to have differing levels of phlebotomy expertise, with those working in July having a particularly high percentage of insufficient specimens.

Four (1.2%) infants out of the 331 infants with birth PCR results tested HIV-positive at birth. Any infant with a positive birth PCR result should be referred to or discussed telephonically with a paediatric HIV specialist for ART initiation. As we found no record-keeping system of the infants’ visits, it was not possible to determine whether ART was initiated in HIV-positive infants. The 2015 national guidelines^7^ state that all PCR results should be documented; however, this information is only recorded in the infant’s Road to Health (RTH) booklet that stays in the mother’s or caregiver’s possession. Only the mother’s details are recorded in the appointment book at the paediatric clinic.

In the authors’ opinion, there is poor communication between the maternity ward and paediatric clinic at MUCPP CHC because the maternity ward was under the impression that the paediatric clinic received the infant PCR results, which was not the case. As a result, none of these departments recorded any information in their files about the PCR results or whether the infant was tested. Thus, the researchers used the NHLS electronic database to get this information. The maternity ward sister indicated to the researchers that the mothers or caregivers were contacted telephonically if the birth HIV PCR was positive.

Of the four infants who tested positive at birth, one was retested at 10 weeks and remained HIV-positive. We speculate that this infant was possibly not on ART and that the birth result was lost, necessitating a repeat PCR. Although the second test could have been confirmatory, the 2015 national guidelines^7^ state that the confirmatory test should be performed within 1 week of a positive birth PCR result.

During the study, we could not find evidence that specific follow-up dates or a general time frame was given to the mother to return for the birth PCR results or repeat testing at 10 weeks. We postulate that mothers were informed at the first vaccination visit at 6 weeks that a repeat PCR should be performed at 10 weeks. A specific date should be given so that the mothers or caregivers know exactly when to come back. According to the national guidelines, 10 weeks is the correct follow-up date because it is essential to do the HIV PCR test 4 weeks after having stopped nevirapine prophylaxis. Nevirapine use could produce a false negative or indeterminate result.^7,9^

Of the mothers or caregivers who brought the infants back for a follow-up PCR test, either before, during or after the 10-week time frame, we speculate that these are the mothers or caregivers who collected the birth PCR results. Possible reasons why the mother or caregiver did not bring the infant back for follow-up PCR testing are lack of transport, lack of money for transport, relocation, forgotten appointments and/or social and family issues.

### Study limitations

It was difficult to find the 10-week results via NHLS. This may be because of the infant’s name, surname or residential address changing from the time of birth.

No further information was captured for the 53 infants who were not tested at birth.

## Conclusion

The researchers found that 87.6% of HIV-exposed infants received a birth HIV PCR test, which approaches the national target of 90.0%. There were no follow-up dates given for infants who tested negative at birth. The mothers or caregivers of those who tested positive at birth were telephonically contacted to return so that initiation of ART could commence, but no record was maintained. The authors speculate that 57.3% of mothers or caregivers who came back for a follow-up test received the birth PCR test results.

Therefore, MUCPP CHC is partially adhering to the 2015 national guidelines^7^ as the majority of HIV-exposed infants are being tested at birth. However, they do not ensure that negative cases are retested to identify intrapartum infection. As only the mothers’ details were recorded at the paediatric clinic, it could not be determined whether the infants who were HIV-positive were initiated on ART or whether a confirmatory PCR test was performed.

The national guidelines^7^ state that all HIV PCR results should be documented. However, this information is recorded in the RTH booklet, which stays with the mothers or caregivers. As of 2017, a record-keeping system was initiated where the clinical staff of the paediatric clinic record the mother’s contact details; however, the infant’s treatment should also be recorded.

### Recommendations

The maternity ward and paediatric clinic at MUCPP CHC should establish a more detailed record-keeping system and improve the communication between the two departments to keep track of testing, results and patient follow-up to provide a continuum of care through the postnatal period. A written date for the 10-week follow-up test should be given on an appointment card to the mother or caregiver. The maternity ward and paediatric clinic should also record that the date was given to the mother or caregiver.

A future study could be conducted to determine whether infants who tested positive at birth had a confirmatory test. In addition, infant follow-up testing through 18 months of age should be documented. Feedback should be given to the clinic and HIV programme. This setting is ideal for a continuous quality improvement programme.^13^
